# Evaluation and analysis of the adsorption mechanism of three emerging pharmaceutical pollutants on a phosphorised carbon-based adsorbent: Application of advanced analytical models to overcome the limitation of classical models

**DOI:** 10.1016/j.heliyon.2024.e34788

**Published:** 2024-07-18

**Authors:** Fatma Dhaouadi, Fatma Aouaini, Beriham Basha, Adrián Bonilla-Petriciolet, Jordana Georgin, Abdelmottaleb Ben Lamine

**Affiliations:** a Laboratory of Quantum and Statistical Physics, LR18ES18, Faculty of Sciences of Monastir, Monastir University, Monastir, Tunisia; b Department of Physics, College of Science, Princess Nourah Bint Abdulrahman University, P.O. Box 84428, Riyadh, 11671, Saudi Arabia; c InstitutoTecnológico de Aguascalientes, 20256, Aguascalientes, Mexico; d Departmentof Civil and Environmental, Universidad de la Costa, CUC, Calle 58 # 55–66, Barranquilla, Atlántico, Colombia

**Keywords:** Sulfamethoxazole, Ketoprofen, Carbamazepine, Double layer adsorption modeling

## Abstract

The double layer adsorption of sulfamethoxazole, ketoprofen and carbamazepine on a phosphorus carbon-based adsorbent was analyzed using statistical physics models. The objective of this research was to provide a physicochemical analysis of the adsorption mechanism of these organic compounds via the calculation of both steric and energetic parameters. Results showed that the adsorption mechanism of these pharmaceuticals was multimolecular where the presence of molecular aggregates (mainly dimers) could be expected in the aqueous solution. This adsorbent showed adsorption capacities at saturation from 15 to 36 mg/g for tested pharmaceutical molecules. The ketoprofen adsorption was exothermic, while the adsorption of sulfamethoxazole and carbamazepine was endothermic. The adsorption mechanism of these molecules involved physical interaction forces with interaction energies from 5.95 to 19.66 kJ/mol. These results contribute with insights on the adsorption mechanisms of pharmaceutical pollutants. The identification of molecular aggregates, the calculation of maximum adsorption capacities and the characterization of thermodynamic behavior provide crucial information for the understanding of these adsorption systems and to optimize their removal operating conditions. These findings have direct implications for wastewater treatment and environmental remediation associated with pharmaceutical pollution where advanced adsorption technologies are required.

## Highlights

• Removal of three emerging pharmaceutical pollutants from aqueous solutions, using **a** phosphorised carbon-based adsorbent.
• Pharmaceutical molecules adsorption mechanism was analyzed via statistical physical modeling to overcome the limitation of classical models.
• The physiochemical parameters of the appropriate model were examined theoretically as a function of temperature.

## 1 Introduction

The widespread implementation of drugs in health services has caused that a significant quantity of these substances can be discharged into the environment via unused or degraded forms [1]. The prevalence of pharmaceutical products in the aquatic environment has been recognized as a major hazard due to the harmful properties of these substances [2]. The continuous exposure of the marine environment to pharmaceuticals pollution can cause significant long-term damage to aquatic life and the people interacting with this ecosystem due to the disruption of the hormone system and the occurrence of susceptible bacterial organisms into the environment [3]. The environmental presence of pharmaceuticals as emerging pollutants has raised significant concerns due to their limited biodegradability, prolonged persistence, and propensity for bioaccumulation. Several categories of drugs have been classed as important environmental pollutants, specifically antibiotics, steroids, antidepressants, antacids, analgesics, antipyretics, beta-blockers, lipid-lowering drugs, tranquilizers, anti-inflammatories, and stimulants [4,5].

Therefore, the removal of these pollutants from aqueous solution and the reduction of their concentrations is of great interest worldwide. In this context, various technologies like biodegradation, electrocoagulation, ozonation, ultrafiltration, membrane-based separation, photocatalytic oxidation and biological processes have been employed to address the pollution caused by pharmaceutical products [[6], [7], [8], [9], [10]]. Several advanced treatment methods are expensive in terms of implementation, operation and maintenance, making them financially prohibitive for some communities. On the other hand, some methods generate harmful by-products, such as toxic sludge, that generate additional disposal challenges and may further contribute to environmental pollution. The complexity of certain treatment processes may require specialized expertise, limiting their applicability in certain regions or communities. The release of secondary pollutants as a result of treatment processes presents an ongoing challenge. These secondary pollutants may be even more hazardous than the original contaminants, exacerbating environmental and public health risks [11]. Thus, it is mandatory to develop reliable and engineered treatment technologies for achieving a sustainable and low-cost depollution of this category of contaminants. The adsorption is currently a leading water treatment method due to its ease of operation, flexibility, reduced generation of waste and by-products, its low energy requirement, besides the adsorbent can be regenerated and reused several times to minimize the water treatment cost [12,13]. Activated carbon, biomass waste, mesoporous silica, zeolite, chitosan, carbon nanotubes, biochar, clays, resin, and graphene oxides are examples of adsorbents that have been successfully utilized to remove pharmaceutical pollutants from wastewater [[14], [15], [16], [17]]. Different studies have proved that the proper functionalization of these materials can contribute to improve their performance for the removal of pharmaceuticals. Particularly, the phosphorization process to functionalize the adsorbent surface has shown several advantages to improve the adsorption properties of carbon-based materials because the functional groups containing P and O have a high affinity to remove the pharmaceutical molecules [18]. For instance, Sekulic et al. (2019) proved that phosphorised carbon-based adsorbents can outperform the pharmaceutical adsorption capacities of activated carbons, multi-walled carbon nanotubes, and biochars. Therefore, these materials are promising for the depollution of water containing pharmaceutical molecules.

The major purpose of this research paper was to investigate, from a theoretical point of view, the adsorption mechanism of emerging contaminants (namely sulfamethoxazole, ketoprofen and carbamazepine) on a phosphorus carbon-based adsorbent (PCA) using advanced physical models developed by the grand canonical ensemble. The steric parameters involved in the adsorption of these compounds were analyzed and discussed including the calculation of the corresponding interaction energies. Modeling results reported in this study contribute to improve the understanding of the removal mechanisms of emerging pollutants with the aim of intensifying the water treatment via adsorption.

## 2 Experimental adsorption isotherms

**Fig. 1:**
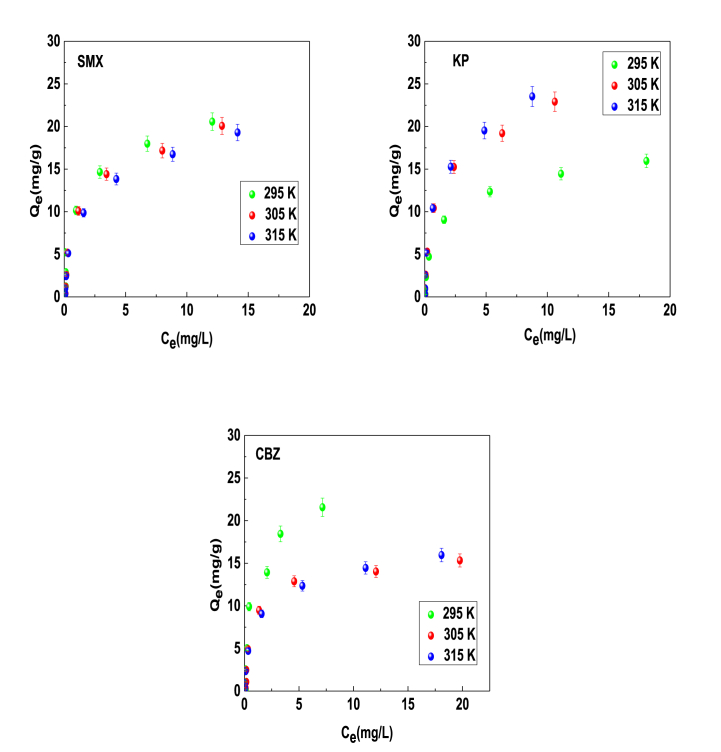
Experimental isotherms of the adsorption of SMX, KP and CBZ molecules on phosphorised carbon-based adsorbent at 295, 305 and 315 K and pH 6 from aqueous solutions.

Fig. 1 displays the experimental data of adsorption isotherms at 295–315 K and pH 6 of sulfamethoxazole (SMX), ketoprofen (KP), and carbamazepine (CBZ) molecules using a phosphorus carbon-based adsorbent (PCA), which was reported in Ref. [18]. These data were obtained under batch conditions at pH 6 using an adsorbent dose of 2 g/L, initial pharmaceutical concentrations of 1–50 mg/L and 140 rpm of stirring speed using a mechanical stirrer (Heidolph Unimax 1010, Germany) to reach the equilibrium after 60 min of contact time. The pharmaceuticals in the aqueous solutions were quantified by HPLC with a diode array detector (High Performance Liquid Chromatography, Agilent 1260 series). The preparation of PCA from the lignocellulosic waste biomass implied several steps. Initially, the biomass was cleaned with ultrapure water and then mechanically ground. Subsequently, the ground biomass was soaked in 50 % H_3_PO_4_ solution, followed by drying at 60 °C for 2 h and carbonization process in a muffle furnace. The first carbonization phase involved the heating of sample at a rate of 10 °C/min until reaching 180 °C. In the second phase, an additional thermal treatment was performed at 500 °C for 1 h, using the same heating rate of 10 °C/min. Finally, the adsorbent was dried at 110 °C for 2 h and homogenized in its particle size to perform the removal experiments of pharmaceuticals [18].

The experimental results depicted the representative monotonic evolution of the equilibrium adsorption capacity (Q_e_) with respect to the equilibrium liquid concentration (C_e_) for the three tested molecules. At high equilibrium concentrations, the adsorption capacities of these emerging pharmaceutical contaminants move towards the saturation value, changing with temperature and achieving the highest value that corresponded to 20.56, 23.52 and 21.56 mg/g for SMX, KP and CBZ, respectively. These saturation conditions reflect the loading of available adsorption sites on the PCA surface and can be associated to the formation of different adsorption adsorbate layers at different temperatures depending on the physicochemical properties of tested pharmaceuticals. It was observed that the impact of temperature on the adsorption capacity was exothermic for SMX and CBZ since an enhancement of the adsorption capacity was noticed as a function of a temperature increment, meanwhile an endothermic adsorption occurred for KP at tested operating conditions. Note that Sekulic et al. (2019) have applied two traditional models, Langmuir and Freundlich, to explain the experimental adsorption profiles of SMX, KP and CBZ on PCA [18]. The application of these classical models represented an initial fundamental step to comprehend the adsorption mechanism of these compounds. However, they may generate erroneous conclusions and interpretations due to the limitations of the background model hypotheses. Consequently, three advanced models developed from statistical physics theory have been utilized to improve the interpretation of the adsorption mechanisms of these systems. These models assume that it is feasible the generation of one, two or more layers of the investigated pharmaceutical molecules on the PCA surface. Note that this type of organic molecules can form aggregates in the aqueous solutions and, consequently, a multilayer adsorption process is feasible. Therefore, these models can be utilized to calculate steric and energetic parameters to obtain a better understanding of these adsorption systems. A brief description of the model implemented in this study is provided below.

## 3 Detailed description of the tested analytical models

**Fig. 2:**
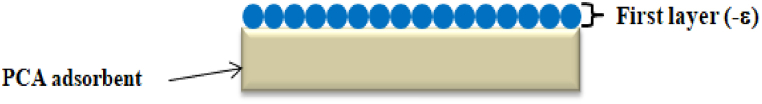
Schematic illustration of the adsorption mechanism associated with PMM.

a) **Physical monolayer model (PMM):** This model recognizes that the surface of PCA adsorbent has only one type of functional group that is involved in the adsorption of SMX, KP, and CBZ molecules. This interface phenomenon implies an adsorption energy between the pharmaceutical molecules and the adsorbent surface. Furthermore, it was assumed that the functional group can adsorb 'n_m_' molecules at the tested adsorption temperatures (295, 305 and 315 K) where a monolayer of adsorbed molecules can be formed as illustrated in Fig. 2.

Accordingly, the adsorbed monolayer quantity of PMM is obtained by the following expression (equation (1)) [19]:

(1) $$Q_{e}=\frac{n_{m}D_{m}}{1+{\left(\frac{C_{1/2}}{C_{e}}\right)}^{n_{m}}}$$

**Fig. 3:**
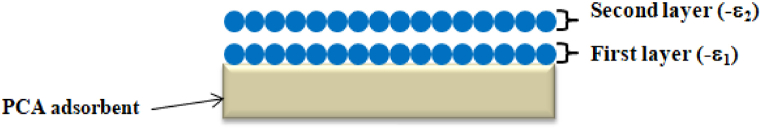
Schematic representations of the formation of two layers on the PCA surface.

b) **Physical double layer model (PDLM):** This second model speculated that the adsorption of pharmaceutical molecules occurred with the formation of two adsorbate layers on the PCA surface. The interactions between the pharmaceutical molecules and the functional groups are described by a first energy (-ε_1_), and a second energy (-ε_2_) also existed for the interactions between the adsorbate molecules that formed the second layer (see Fig. 3).

In fact, the equilibrium adsorbed quantity of the molecules as a function of the equilibrium adsorbate concentration is represented by equation (2) [20]:

(2) $$Q_{e}=n_{m}D_{m}\frac{{\left(\frac{C_{e}}{C_{1}}\right)}^{n_{m}}+2{\left(\frac{C_{e}}{C_{2}}\right)}^{2n_{m}}}{1+{\left(\frac{C_{e}}{C_{1}}\right)}^{n_{m}}+{\left(\frac{C_{e}}{C_{2}}\right)}^{2n_{m}}}$$

**Fig. 4:**
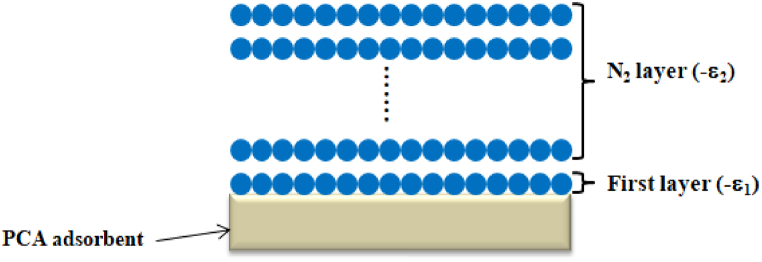
Illustration of adsorption mechanism based on AMPM model.

c) **Advanced multilayer physical model (AMPM):** This model is a general case in which it was hypothesized that the adsorption of pharmaceutical molecules occurred via the formation of a variable number of layers where two interaction energies were involved. As in the double layer model, these adsorption energies are associated with the interactions of the first and other supplementary layers of adsorbed pharmaceutical molecules, respectively. It is noted that the total number of formed pharmaceutical layers was identified as 1 + N_2_ in this model (Fig. 4).

The functionality of the adsorbed quantity with respect to the equilibrium pharmaceutical concentration is provided by equation (3) [21]:

**Figure:**
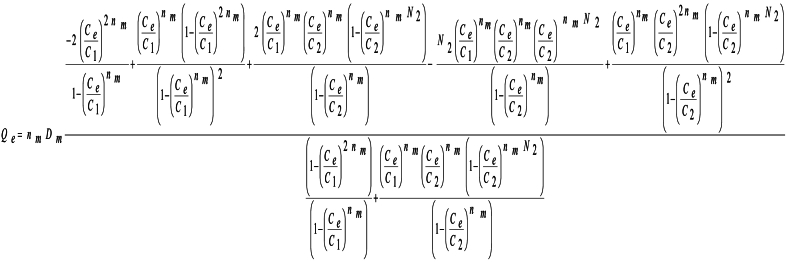

In these three models, the parameter n_m_ is the number of pharmaceutical molecules adsorbed by the PAC functional group, D_m_ is the density of this functional group on the adsorbent surface (mg/g), C_1_ is the half-saturation concentration of the first pharmaceutical layer formed on the PCA surface (mg/L), C_2_is the half-saturation concentration associated with the formation of N_2_ pharmaceutical layers (mg/L) with the second interaction energy and, finally, the total number of adsorbed pharmaceutical layers is defined by N_L_ = 1 +N_2_ where N_2_ = 0 and = 1 in the monolayer and double-layer models, respectively.

These statistical physics adsorption models were implemented to fit all the experimental isotherms to forecast the expected number of formed layers of SMX, KP, and CBZ molecules adsorbed on the PCA surface via the adsorption process, the number of adsorbed pharmaceutical molecules per functional group and the density of functional groups involved in the pharmaceutical adsorption, the saturation adsorption capacities, and the related adsorption energies. The Levenberg-Marquardt algorithm was used to carry out the mathematical fitting of the experimental data via a multivariate nonlinear regression. A comparison of these models was performed and the best model was selected using the coefficient of determination (R^2^) and a global analysis of the evolution and trend of each model's parameters. R^2^ was calculated with equation (4) [22]:

(4) $$R^{2}=1-\left[1-{\left(\frac{\sum \left(x_{i}-\bar{x}\right)\;\left(y_{i}-\bar{y}\right)}{\sqrt{\sum {\left(x_{i}-\bar{x}\right)}^{2}}*\sqrt{\sum {\left(y_{i}-\bar{y}\right)}^{2}}}\right)}^{2}\right]\left[\frac{n_{p}-1}{d_{f}}\right]$$

where n_p_ is the number of experimental adsorption data, d_f_ is the number of degrees of freedom of each adsorption model, $\bar{x}$ and $\bar{y}$ are the average experimental and preferred values of x_i_ and y_i_, respectively. For illustration, Table 1 summarizes the coefficients of determination (R^2^) for the tested adsorption models.

**Table 1:** Results of isotherm data modeling of the adsorption of SMX, KP and CBZ molecules on PCA surface at pH 6 using statistical physics models.

T (K)	R^2^ for		
	SMX	KP	CBZ
PMM			
**295**	0.949	0.989	0.936
**305**	0.978	0.987	0.978
**315**	0.985	0.984	0.989
**R**^**2**^_**average**_	0.970	0.986	0.967
**PDLM**			
**295**	0.937	0.994	0.983
**305**	0.990	0.992	0.980
**315**	0.994	0.983	0.994
**R**^**2**^_**average**_	0.973	0.989	0.985
**AMPM**			
**295**	0.928	0.992	0.977
**305**	0.987	0.993	0.985
**315**	0.994	0.989	0.992
**R**^**2**^_**average**_	0.969	0.991	0.984

**Fig. 5:**
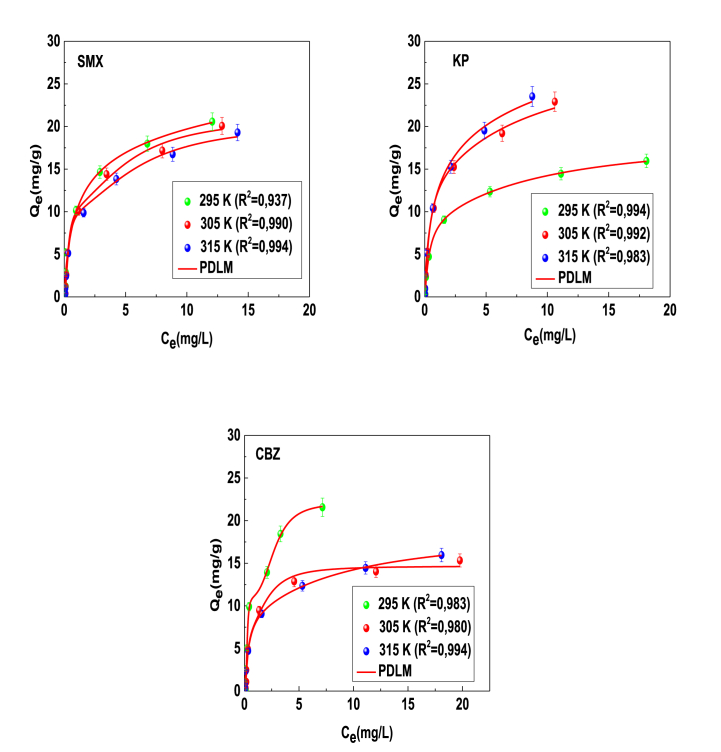
Data fitting results of PDLM model for the experimental isotherms of SMX, KP and CBZ molecules on the PCA surface. R^2^ is the coefficient of determination.

An analysis of the parameters of each model and R^2^ values suggested that the double layer model, which hypothesized the formation of two layers of the adsorbed pharmaceutical molecules on the PCA surface, was adopted to explain the adsorption mechanism of the tested systems. The estimated parameters and fitting curves of the adsorption data are provided in Table 2 and Fig. 5.

**Table 2:** Physicochemical parameters associated with the PDLM model estimated by fitting of adsorption isotherms of pharmaceutical molecules.

T(K)	n_m_	D_m_ (mg/g)	C_1_ (mg/L)	C_2_ (mg/L)
SMX				
**295**	0.84	21.58	0.89	7.03
**305**	1.98	5.33	0.31	1.26
**315**	1.95	5.22	0.32	1.39
**KP**				
**295**	1.22	7.67	0.35	1.80
**305**	1.18	12.16	0.36	1.81
**315**	0.81	22.36	0.63	3.37
**CBZ**				
**295**	3.38	3.25	0.24	0.81
**305**	2.22	3.30	0.27	0.76
**315**	1.22	7.67	0.35	1.80

In order to provide a detailed explanation of the adsorption mechanism of tested pharmaceutical molecules on the functional groups of the PCA surface, all the adjusted parameters of the double layer model with two energies were analyzed and discussed as a function of the adsorption temperature.

## 4 Discussion, examination and explanation of the physicochemical parameters obtained with the statistical physics theory

### 4.1 Impact of thermal agitation on steric parameter “n_m_”

The parameter n_m_ is a relevant stoichiometric coefficient that can be used to analyze the stereography (i.e., geometry) of the adsorption mechanism and it is also important to identify the type of anchorage between the pharmaceutical molecules and the adsorption sites. In addition, it offers information about the aggregation phenomenon of pharmaceutical molecules in the aqueous solution. As an illustration, there are three scenarios depending on the value of n_m_ [20]:

If n_m_ < 0.5: it is the case of a parallel anchoring where the pharmaceutical molecules can interact with two or more adsorption sites.

If 0.5 < n_m_ < 1: the pharmaceutical molecules can be adsorbed via a combined interaction (i.e., interaction through one and two adsorption sites is feasible but with two different proportions).

If n_m_ > 1: the adsorption mechanism is multimolecular where each functional group can receive several molecules simultaneously.

**Fig. 6:**
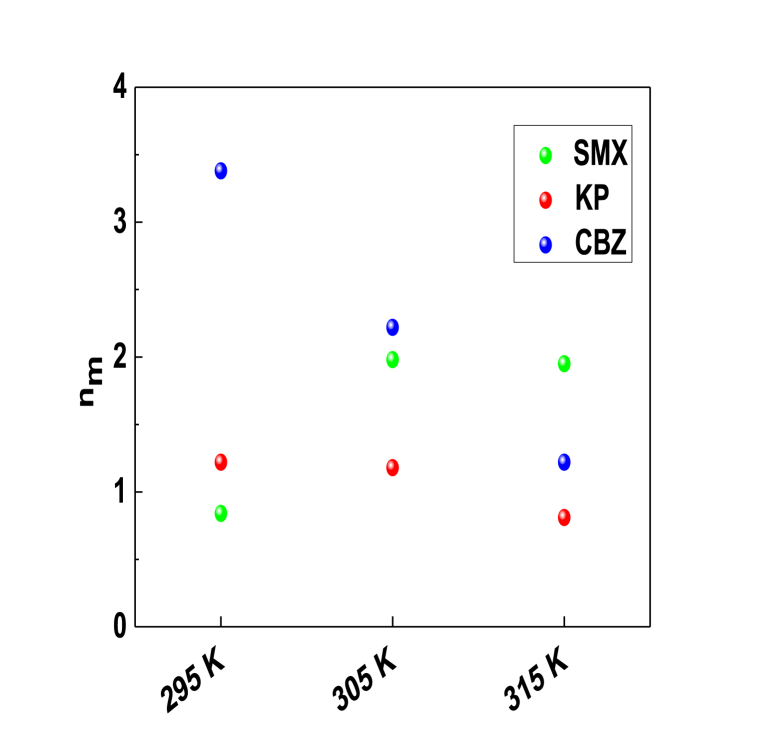
n_m_ versus temperature for the adsorption of SMX, KP and CBZ molecules on PCA surface at 295–315 K and pH 6 from aqueous solutions.

Fig. 6 represents the effect of temperature on the number of SMX, KP, and CBZ molecules adsorbed per functional group from PCA surface.

Table 2 and Fig. 6 showed that the calculated values of the number of SMX, KP and CBZ molecules adsorbed per functional group (n_m_) were higher than unity with the exception of two cases (i.e., n_m_ = 0.84 at 295 K for SMX molecules, and n_m_ = 0.81 at 315 K for KP molecules). The dominant scenario (n_m_ > 1) indicated that the adsorption mechanism of these pharmaceutical molecules was multimolecular where each functional group from the PCA surface can adsorb several molecules simultaneously. For the two exceptional cases, the anchoring of the molecules on the adsorption sites was a mixed type (i.e., parallel and non-parallel anchoring) where the percentage of interaction with one site was greater than the interaction with two adsorption sites since 0.75 < n_m_ < 1. Thermally speaking, the number of KP and CBZ molecules captured per functional group decreased as a function of solution temperature, thus suggesting that the molecular aggregation phenomenon in the solution was exothermic where the temperature caused the rupture of bonds between the pharmaceutical molecules. In contrast, the thermal agitation favored the aggregation between the SMX molecules and this process was endothermic where the presence of monomers and dimers in the aqueous solution is expected.

### 4.2 Analysis of D_m_ and Q_sat_ parameters

**Fig. 7:**
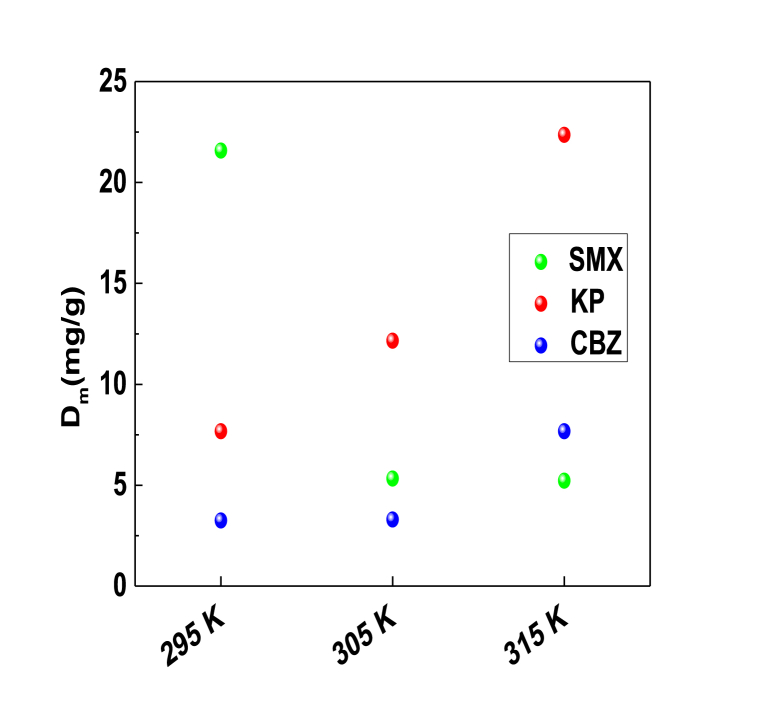
The density of functional group D_m_ versus temperature for the adsorption of SMX, KP and CBZ molecules on PCA surface at 295–315 K and pH 6 from aqueous solutions.

Fig. 7 plots the impact of adsorption temperature on the variation of the functional group density D_m_ for tested pharmaceuticals.

Fig. 7 illustrated that the temperature enhanced the functional group density for the adsorption of both KP and CBZ molecules, while the temperature had a negative effect on this steric parameter (D_m_) for the case of SMX molecules. These observations demonstrated that the adsorption temperature showed an antagonistic impact to that identified for the n_m_ parameter. Actually, the decrement of the n_m_ parameter produced an eventual increase in the number of anchoring sites, then an expected growth in the functional group density and vice versa.

Based on these two steric parameters (i.e., n_m_ and D_m_), the adsorbed quantity at saturation can be estimated using equation (5):

(5) $$Q_{sat}=2\times n_{m}\times D_{m}$$

**Fig. 8:**
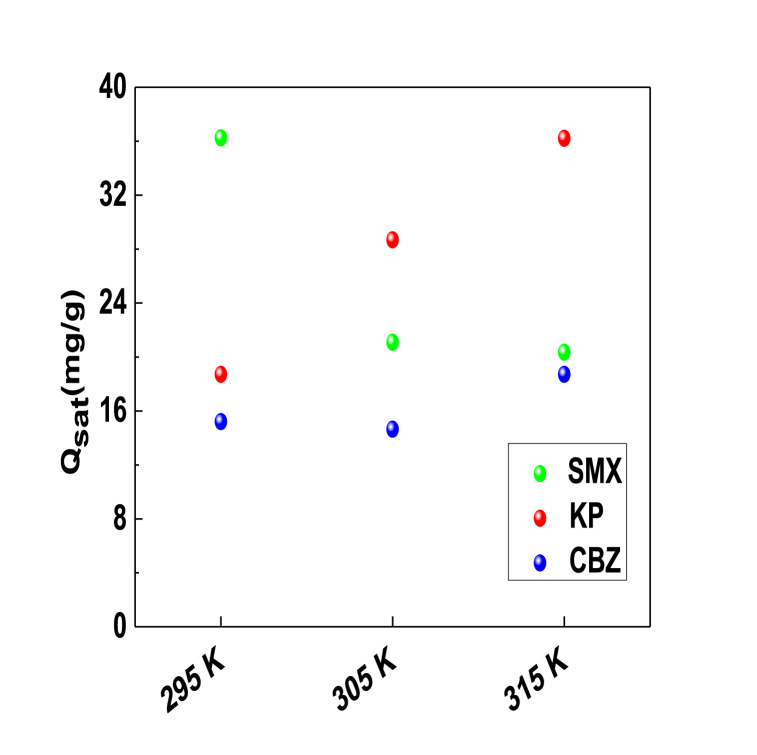
Temperature dependency of the adsorbed quantities at saturation for the SMX, KP and CBZ pharmaceutical molecules.

The influence of temperature on this saturation adsorption capacity is depicted in Fig. 8.

Adsorption temperature caused two behaviors on the performance of PCA adsorbent to remove pharmaceutical molecules. The adsorbed quantities at saturation of KP and CBZ molecules increased with the temperature rise thus achieving 36.22 and 18.71 mg/g, respectively. These findings demonstrated that the thermal agitation caused that more active sites participated in the adsorption of these molecules, thus resulting in the improvement of adsorbent performance. In contrast, the adsorbed quantity at saturation for SMX molecules decreased with the temperature increment suggesting that thermal agitation may cause the inactivation of some functional groups responsible of the adsorption process. To conclude, the adsorbed quantities of these tested pharmaceutical molecules (SMX, KP and CBZ) were governed by the density of functional groups D_m_ since this parameter increased with temperature.

### 4.3 Analysis of the adsorbent-adsorbate and adsorbate-adsorbate interactions

The statistical physics model can be utilized to calculate the energy of the interactions between the functional groups of PCA and the pharmaceutical molecules, and between the pharmaceutical molecules, using the next generalized equation (6) [23]:

(6) $$\left(-\varepsilon_{1,2}\right)=RT\;\ln\left(\frac{C_{s}}{C_{1,2}}\right)$$

where Cs is the solubility of the pharmaceutical molecules in water and R is the ideal gas constant. Table 3 reports the calculated adsorption energies at 295–315 K and pH 6 for tested systems.

**Table 3:** Calculated adsorption energies for the adsorption of pharmaceutical molecules on the PCA surface at different temperatures and pH 6.

T (K)	$\left(-\varepsilon_{1}\right)$ (kJ/mol)	$\left(-\varepsilon_{2}\right)$ (kJ/mol)
**SMX**		
**295**	15.95	10.89
**305**	19.14	15.59
**315**	19.66	15.83
**KP**		
**295**	12.20	8.19
**305**	12.53	8.44
**315**	11.46	7.09
**CBZ**		
**295**	10.53	7.55
**305**	10.57	7.95
**315**	10.23	5.95

It can be seen that all the values of calculated adsorption energies were lower than 40 kJ/mol and it was concluded that the adsorption of tested pharmaceutical molecules by PCA involved physical interaction forces. In particular, π−π interactions and/or hydrogen bonding may be implicated in the adsorption mechanism of SMX, KP and CBZ molecules using this adsorbent. Note that the interaction energies of SMX were higher than those obtained for the two other molecules (KP and CBZ), thus reflecting a stronger interface interaction due to the different structures of the pharmaceutical molecules.

Herein, it is convenient to indicate that the statistical physics models are not able to identify the chemical nature of the adsorption sites involved in the mechanism to remove tested pollutants. In this case, DFT calculations are required to complement the results obtained from these models with the aim of finalizing the theoretical analysis of these adsorption systems.

### 4.4 Performance comparison of different adsorbents used in the removal of pharmaceutical molecules

Table 4 provides some results published in the literature to compare the adsorption of SMX, CBZ and KP molecules on several carbon-based adsorbents.

**Table 4:** Performance comparison of different adsorbents regarding the maximum adsorption capacity of pharmaceutical molecules.

Pharmaceutical molecules	Adsorbents	Q_max_ (mg/g)	Reference
**SMX**	PCA	36.25	This work
**SMX**	Multi-walled carbon nanotube	28.88	[24]
**SMX**	AC from paper mill sludge	1.39	[18]
**CBZ**	PCA	18.71	This work
**CBZ**	Chemically activated biochar	0.42	[25]
**CBZ**	Raw materials	16.5	[26]
**KP**	PCA	36.22	This work
**KP**	AC from olive-waste cake	24.70	[27]
**KP**	SrCoO_3-δ_	28.40	[28]

These results indicated that the phosphorised carbon-based adsorbents outperformed the adsorption capacity of other adsorbents reported for the removal of these pharmaceutical molecules. This outcome suggested that the tested adsorbent may be a valuable option for the removal of pharmaceutical molecules to minimize the costs implied in the treatment of wastewater from diverse industries.

## 5 Conclusions

A theoretical analysis of the mechanism of adsorption of sulfamethoxazole, ketoprofen and carbamazepine molecules on a phosphorus carbon-based adsorbent was performed using statistical physics theory. Calculations showed that the adsorption of these pharmaceutical molecules implied the formation of a double layer on adsorbent surface. The presence of molecular aggregates in the aqueous solution for these pharmaceuticals was also feasible especially for carbamazepine and sulfamethoxazole. The adsorption of these pharmaceutical was associated with physical interaction forces. An endothermic behavior was observed for the removal of carbamazepine and sulfamethoxazole, while the ketoprofen adsorption was exothermic. The results of this study should be complemented with computational chemistry calculations to characterize the chemical nature of adsorption sites involved in the removal of tested pollutants. This study contributes with new insights on the adsorption mechanism of pharmaceuticals molecules, which are relevant and priority pollutants in water treatment.

## Ethical approval

Not applicable.

## Consent to participate

Not applicable.

## Consent to publish

Not applicable.

## Funding

Not applicable.

## Availability of data and materials

Note applicable.

## CRediT authorship contribution statement

**Fatma Dhaouadi:** Writing – original draft, Formal analysis, Conceptualization. **Fatma Aouaini:** Writing – original draft, Formal analysis, Conceptualization. **Beriham Basha:** Writing – review & editing. **Adrián Bonilla-Petriciolet:** Writing – review & editing. **Jordana Georgin:** Software, Formal analysis. **Abdelmottaleb Ben Lamine:** Writing – review & editing, Supervision.

## Declaration of competing interest

The authors declare that they have no known competing financial interests or personal relationships that could have appeared to influence the work reported in this paper.
